# MMP-2 and MMP-9 Gene Polymorphisms and Serum Levels in Relation to Insulin Resistance in a Polish Cohort

**DOI:** 10.3390/ijms27052158

**Published:** 2026-02-25

**Authors:** Beata Gajewska, Mariola Śliwińska-Mossoń, Helena Moreira

**Affiliations:** 1 Students Scientific Society of Flow Cytometry and Biomedical Research, Faculty of Pharmacy, Wroclaw Medical University, 50-556 Wroclaw, Poland; beata.gajewska@student.umw.edu.pl; 2 Laboratory Diagnostics Department, Lower Silesian Center for Oncology, Pulmonology and Hematology, 53-413 Wroclaw, Poland; mariola.sliwinska-mosson@dcopih.pl; 3 Department of Basic Medical Sciences, Faculty of Pharmacy, Wroclaw Medical University, 50-556 Wroclaw, Poland

**Keywords:** metalloproteinase, insulin resistance, polymorphism

## Abstract

Insulin resistance is a vital component in the diagnosis of prediabetes. Understanding the factors that influence its development and the course of metabolic disorders should include individual genetic predispositions, which may provide insight into etiology and potential prevention strategies. In this study, we examined associations between MMP-2 −1306 C/T (rs243865) and MMP-9 −1562 C/T (rs3918242) single-nucleotide polymorphisms, serum levels of MMP-2, MMP-9 and TIMP-1, and the occurrence of insulin resistance in patients in a Polish cohort. DNA isolated from 200 unrelated individuals was studied; participants were divided into insulin-resistant and control groups based on the homeostatic model assessment criteria (HOMA-IR). Genotyping of MMP-2 −1306 C/T (rs243865) and MMP-9 −1562 C/T (rs3918242) SNPs was performed using polymerase chain reaction (PCR) and restriction fragment length polymorphism (RFLP) techniques. Serum MMP-2, MMP-9, and TIMP-1 concentrations were measured by enzyme-linked immunosorbent assays (ELISA). No significant associations were observed between the investigated MMP-2 and MMP-9 polymorphisms or TIMP-1 concentrations and the occurrence of insulin resistance. MMP-9 exhibited genotype-related variation, and MMP-2 concentrations differed between the IR and CTR groups. No significant correlations were found between MMP-2 or MMP-9 and TIMP-1. Our data do not support a direct association between the analyzed polymorphisms and insulin resistance in this sample. The observed higher MMP-2 levels in controls warrant further study. Larger, multi-ethnic studies are required to confirm these findings.

## 1. Introduction

Insulin resistance (IR) is a pathological state that may precede prediabetes and type 2 diabetes (T2D). The principal mechanism involves reduced sensitivity of insulin-responsive tissues (primarily adipose and skeletal muscle) to insulin action [1]. Consequently, intracellular glucose uptake decreases, leading to elevated blood glucose concentrations; this stimulates pancreatic insulin secretion and may exacerbate cellular insulin resistance [2]. Unchecked, this vicious cycle can result in insufficient insulin secretion under persistent hyperglycemia and progression to T2D, a chronic disease associated with multiple long-term complications [3]. Therefore, identifying factors that predispose to IR—and potentially intercepting progression at this stage—is clinically important to avoid serious health consequences [4].

However, it is important to examine all factors that may influence individual predispositions to metabolic disorders. In our study, we focused on single-nucleotide polymorphisms of genes (SNPs) encoding the family of extracellular matrix metalloproteinases (MMPs)—gelatinases. The role of MMPs in insulin resistance is closely linked to the remodeling of the extracellular matrix (ECM) within adipose tissue. In the state of chronic overnutrition, rapid expansion of adipocytes requires dynamic ECM degradation, a process primarily mediated by gelatinases, MMP-2 and MMP-9, which under physiological conditions are responsible for tissue remodeling, angiogenesis, and embryonic development [5,6]. Failure or dysregulation of this remodeling leads to adipose tissue fibrosis, which triggers local hypoxia and chronic low-grade inflammation—key drivers of impaired insulin signaling. Furthermore, MMP-9 has been implicated in the shedding of the insulin receptor from the cell surface, directly contributing to decreased insulin sensitivity. While excessive activity of these enzymes may participate in the development of pathological conditions and diabetic complications such as angiopathy, nephropathy, and diabetic foot, clinical evidence regarding circulating MMP levels and their genetic determinants in prediabetic states remains inconsistent [7,8,9,10]. This justifies the need to investigate whether differences in specific polymorphisms, such as MMP-2 −1306 C/T (rs243865) and MMP-9 −1562 C/T (rs3918242), affect susceptibility to insulin resistance, as suggested by previous reports [7,8,9,10,11].

Considering MMP-2 and MMP-9 concentration measurements when discussing the effects of the analyzed polymorphisms is particularly important, as the mechanisms through which these genetic variants exert their influence relate directly to alterations in gene expression. The molecular consequences of the presence of T alleles in both cases arise from the localization of the variants in promoter regions. The MMP-2 −1306 C/T (rs243865) polymorphism reduces promoter activity through a C/T substitution that disrupts the Sp1 binding site, leading to decreased transcription of MMP-2 [12,13,14]. In contrast, the MMP-9 −1562 C/T (rs3918242) polymorphism decreases the binding of a nuclear repressor protein, resulting in increased promoter activity and elevated transcription of MMP-9 [15,16]. Neither polymorphism alters the amino-acid sequence of the encoded enzymes; their impact relies solely on modifications in gene expression. Therefore, incorporating assessments of MMP-2 and MMP-9 levels into this study, alongside genotypic analyses, appears to be fully justified. This approach provides a comprehensive perspective—from genomic alteration, through its molecular effects, to potential clinical implications.

The biological activity of matrix metalloproteinases is not determined solely by their concentration but by the dynamic balance between these enzymes and their endogenous inhibitors, primarily TIMP-1 [17]. TIMP-1 not only regulates extracellular matrix remodeling but also acts as a signaling molecule in inflammatory pathways and adipogenesis. Therefore, evaluating TIMP-1 levels is essential to understanding the overall proteolytic environment in the context of insulin resistance. As an inhibitor of both MMP-2 and MMP-9, TIMP-1 may exert a potential protective effect against the occurrence of disorders associated with their excessive activity, which is why we incorporated the examination of its systemic levels into our study design [17].

## 2. Results

The analysis of biochemical parameters showed that the studied IR and CTR groups were matched in terms of gender distribution (*p* = 0.5525), total cholesterol level (*p* = 0.6702) and LDL level (*p* = 0.9764).

There were no statistically significant relationships between the frequency of individual genotypes or for either polymorphism and whether patients belonged to the insulin resistance group or the control group. In the case of the MMP-2 −1306 C/T SNP (rs243865), the *p*-value for individual genotypes was CT versus CC (*p* = 0.5993), TT versus CC (*p* = 0.6509), TT versus CC + CT (*p* = 0.7063), CT + TT versus CC (*p* = 0.5470); for alleles: T versus C (*p* = 0.5307).

In the case of the MMP-9 −1562 C/T (rs3918242) SNP, the *p*-value for individual genotypes was CT versus CC (*p* = 0.2818), TT versus CC (*p* = 0.7989), TT versus CC + CT (*p* = 0.7063), CT + TT versus CC (*p* = 0.3724); for alleles: T versus C (*p* = 0.5287). (Table 1).

The distribution of genotypes for both MMP-2 and MMP-9 was in Hardy–Weinberg equilibrium (MMP-2 IR: *p* = 0.8964, CTR: *p* = 0.9295; MMP-9 IR: *p* = 0.9858, CTR: *p* = 0.4066).

The results of the measurements of MMP-2 and MMP-9 levels are presented collectively in Table 2, with a breakdown by clinical status regarding the presence of insulin resistance as well as by specific genotype.

A comparison between the group of patients with insulin resistance and the control group revealed a statistically significant difference in MMP-2 levels (*p* = 0.0227), with the control group exhibiting higher mean values than the IR group. No analogous relationship was observed for MMP-9 (*p* = 0.2411).

**Figure 1:**
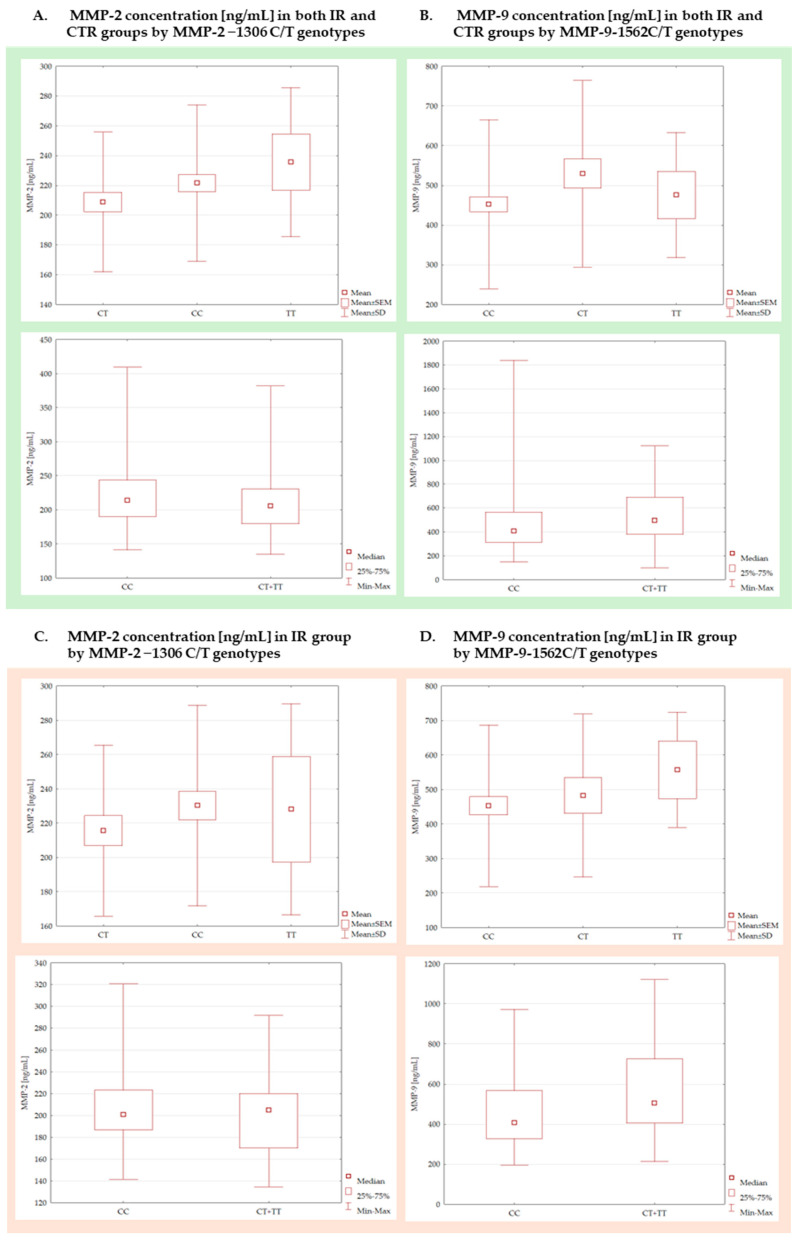

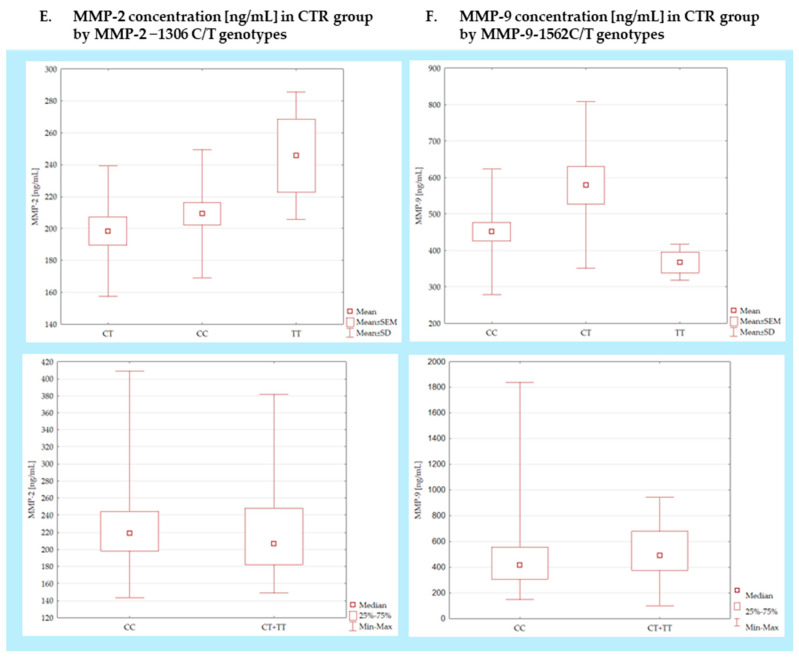
Comparison of MMP-2 and MMP-9 concentrations in the general group (**A**,**B**), IR (**C**,**D**) and CTR (**E**,**F**) divided into genotypes of MMP-2 −1306 C/T polymorphism (**A**,**C**,**E**) and MMP-9 −1562 C/T polymorphism (**B**,**D**,**F**). The *p* values for the Kruskal–Wallis test—CC vs. CT vs. TT were (**A**) (*p* = 0.1426), (**B**) (*p* = 0.0985), (**C**) (*p* = 0.0884), (**D**) (*p* = 0.3553), (**E**) (*p* = 0.4244), (**F**) (*p* = 0.0398). For the Mann–Whitney U test—CC vs. CT + TT the *p* values were (**A**) (*p* = 0.2246), (**B**) (*p* = 0.0349), (**C**) (*p* = 0.7518), (**D**) (*p* = 0.0638), (**E**) (*p* = 0.2059), (**F**) (*p* = 0.2734).

Based on the statistical analysis of these data, no statistically significant differences in MMP-2 concentrations were observed among the CC, CT, and TT genotypes in either the group of patients with insulin resistance (IR: *p* = 0.0884) or the control group (CTR: *p* = 0.4244). Likewise, no statistically significant effect of the presence of the T allele within the genotype was identified in these groups: IR (*p* = 0.7518) and CTR (*p* = 0.2059). In the case of MMP-9 concentrations, a statistically significant difference was found within the insulin-resistant group between the CT and CC genotypes (*p* = 0.0398), with the CT genotype exhibiting higher mean values. No such associations were detected in the control group (*p* = 0.3553). The analysis of the impact of the T allele also revealed a statistically significant difference in the overall sample (*p* = 0.0349), although this effect did not remain significant when examined separately within the IR (*p* = 0.0637) and CTR (*p* = 0.2764) subgroups. All these analyses are illustrated using box plot graphs for both metalloproteinases (Figure 1).

The correlation analysis between metalloproteinase concentrations and their inhibitor TIMP-1 showed no statistically significant associations either in the group of patients with insulin resistance (IR: MMP-2 *p* = 0.2728; MMP-9 *p* = 0.2809) or in the control group (CTR: MMP-2 *p* = 0.3474; MMP-9 *p* = 0.3184).

Analysis of TIMP-1 level results did not show any statistically significant difference between healthy patients and patients with insulin resistance for any of the subgroups in both cases of polymorphism. The *p* value was for MMP-2: CC (*p* = 0.6879), CT (*p* = 0.5055), TT (*p* = 0.5147), and for MMP-9: CC (*p* = 0.7676), CT (*p* = 0.3676), TT (*p* = 0.3335). (Table 3.)

## 3. Discussion

Research on the impact of polymorphisms on metabolic disorders is a crucial factor that brings us closer to understanding the exact mechanisms that control them, including the genetic predispositions of individual patients. Therefore, our study focused on a disorder that in many cases is the beginning of metabolic problems and a precursor to type 2 diabetes. So far, no study has been published linking the polymorphisms described here with insulin resistance, and due to evidence pointing to their association with T2D, they were selected for analysis in this study. Andjelic et al. demonstrated in a group of 102 patients the association of MMP-2 −1306 C/T (rs243865) with type 2 diabetes and related diabetic polyneuropathy. However, they did not find such a relationship in the case of MMP-9 −1562 C/T (rs3918242) [18]. Completely opposite results were presented in two studies by Buraczyńska et al.; their studies were conducted on much larger groups of patients (both on Caucasians of Polish origin), 1090 in the case of rs243865 and 740 in the case of rs3918242. Genotyping of the T2D group and the control group did not show an association of the above-mentioned SNP of MMP-2 with diabetes, but its importance in the occurrence of cardiovascular comorbidity was found [8]. As for the discussed SNP of MMP-9, genotyping indicates the influence of the T allele on both T2D and cardiovascular disease [9]. This result regarding the association with diabetes is confirmed by another study on a group of 730 patients (North Indian population); Singh et al. presented the association of rs3918242 with T2D and its potential importance in the development of diabetic foot ulcer [11]. Genotyping of patients for rs3918242 polymorphism in 310 patients (Han Chinese population) by Feng et al., however, shows a statistically significant protective effect of the TT genotype in the control group compared to the group of diabetic patients. Additionally, the presence of the T allele was associated with a reduced risk of diabetic nephropathy [19]. A similar tendency is shown by Sarray et al. in a study on 791 patients (Tunisian Arab population), where this polymorphism was associated with a reduced risk of developing T2D [7]. Both polymorphisms in a broader context were presented by Yadav et al. in two separate publications. Both rs243865 and rs3918242 (both in groups of 370 patients—Asian Indian population) were described as associated with the occurrence of metabolic syndrome—which is closely related to insulin resistance [20,21].

The results of our studies, both in the case of MMP-2 and MMP-9, do not show any association of individual SNPs with the occurrence of insulin resistance in patients. There was no effect of specific genotypes or the presence of alternative alleles. However, the limitations that influenced this study must be considered. Scientific research on SNPs requires considering the genetic variability of the population on which the work is conducted, which may result in differences in the results. The alternative allele frequency for rs243865 in our study was 0.225 among all participants. This value is lower than the average for the European population, i.e., 0.24467, but higher than the global average—0.20135. In the case of rs3918242, this value was 0.1575, which is lower than the average for the European population, which in this case is 0.16158, and the global population—0.17402 [22]. Another factor that should be taken into account is the small number of patients whose samples were genotyped, which may also affect the results obtained.

Our multivariate analysis confirms the critical role of obesity and dyslipidemia in the development of insulin resistance. Lack of statistical significance for MMP-2 and MMP-9 SNPs in the adjusted model indicates that their contribution is likely modulated by, or secondary to, metabolic factors such as BMI and hypertriglyceridemia. This aligns with the complex polygenic nature of prediabetes, where environmental and metabolic pressures often outweigh the effect size of single-nucleotide polymorphisms.

In our cohort, significantly higher MMP-2 concentrations were observed in the control group compared to individuals with insulin resistance. While some reports [23] have similarly observed lower MMP-2 levels in patients with metabolic disturbances, this finding should be interpreted with caution. Rather than suggesting a direct protective role, the lower MMP-2 levels in the IR group might reflect a complex dysregulation of extracellular matrix (ECM) remodeling in the early stages of metabolic dysfunction. It is possible that the decrease in circulating MMP-2 in insulin-resistant patients is a secondary phenomenon related to altered tissue-specific expression or increased consumption/sequestration of the enzyme within the remodeling adipose tissue or vascular wall. Given that MMP-2 is frequently associated with vascular inflammation and metabolic complications in other contexts, our observation may indicate a state-specific depletion or downregulation rather than a primary protective mechanism. Further functional studies are necessary to determine whether these differences are a cause or a consequence of the insulin-resistant state.

While the functional nature of the MMP-2 −1306 C/T polymorphism suggests that the T allele should lead to reduced promoter activity and lower enzyme levels, our results did not confirm this trend. This inconsistency suggests that in the context of insulin resistance, circulating MMP-2 levels are likely governed by a complex interplay of factors that may override the primary genetic signal. First, metabolic status appears to be a dominant regulator. Our IR group was characterized by significantly higher BMI and triglyceride levels, factors known to promote a pro-inflammatory environment. Chronic low-grade inflammation can stimulate MMP-2 expression through alternative pathways, potentially masking the transcriptional deficit associated with the T allele. Second, epigenetic regulation, such as DNA methylation of the MMP-2 promoter or differential microRNA expression, may further modulate gene output regardless of the underlying SNP. Finally, environmental influences, including diet and physical activity, which were not fully controlled in this study, could contribute to the observed variability. It is also important to note the low frequency of the TT genotype in our cohort (*n* = 8), which limits the statistical power to detect subtle genotype-dependent differences in protein concentration.

A notable finding in our study is the apparent discrepancy between the genetic effect of the MMP-9 -1562 C/T polymorphism and clinical outcomes. While the presence of the T allele was significantly associated with higher circulating MMP-9 levels, consistent with the functional nature of this SNP where the C/T transition disrupts a transcriptional repressor binding site, this did not translate into a significant difference in MMP-9 concentrations between the IR and CTR groups. This observation suggests that while the genetic variant sets a higher baseline for MMP-9 expression, the actual clinical manifestation of insulin resistance is dominated by more potent metabolic drivers. Our multivariate analysis supports this hypothesis, demonstrating that BMI and triglyceride levels are predictors of IR status. In a state of chronic low-grade inflammation associated with obesity, MMP-9 expression may be maximally stimulated by pro-inflammatory cytokines regardless of the underlying genotype, effectively ‘masking’ the genetic contribution of the rs3918242 polymorphism.

In our study, serum TIMP-1 levels did not differ significantly between the IR and CTR groups, nor were they influenced by the investigated MMP-2 and MMP-9 genotypes. This lack of significance is noteworthy, as it suggests that the proteolytic imbalance observed in prediabetes may be driven primarily by fluctuations in enzyme levels (like MMP-2) rather than by a compensatory response from its inhibitor. No significant correlations were identified between MMP-2 or MMP-9 and their inhibitor TIMP-1, suggesting that the observed changes in metalloproteinase levels may not directly translate into overall serum concentrations of the inhibitor, and that the inhibitory mechanism is regulated by more complex processes. However, it is also possible that systemic TIMP-1 levels do not fully reflect local tissue-specific concentrations. In adipose tissue, TIMP-1 is known to be upregulated in obesity and may promote fibrosis, which impairs insulin sensitivity. Our finding of stable systemic TIMP-1 levels, despite higher BMI in the IR group, might indicate that at this early stage of metabolic disturbance (prediabetes), the systemic inhibitory capacity remains relatively preserved. This further emphasizes that the decreased MMP-2/TIMP-1 ratio in the IR group is a result of reduced MMP-2 availability rather than TIMP-1 overproduction.

The present study has certain limitations that should be acknowledged. First, the sample size (*n* = 88 for IR and *n* = 112 for controls) may provide limited statistical power to detect modest genetic associations, particularly for the homozygous rare genotypes (TT) of the MMP-2 rs243865 and MMP-9 rs3918242 polymorphisms, which were present at low frequencies in our cohort. Consequently, our findings regarding the lack of independent genetic associations should be considered preliminary. While this study provides valuable insights into the Polish population, larger-scale studies with greater sample sizes are necessary to definitively establish the role of these metalloproteinase variants in the pathogenesis of insulin resistance.

## 4. Materials and Methods

### 4.1. Study Subjects

The described research was conducted on biological material obtained thanks to the BioBank Research Group of the Łukasiewicz Research Network—PORT (Wroclaw, Poland) and its use has been approved by the local research and ethics committees. Whole blood was collected from 200 unrelated adults of uniform Polish ethnicity, different age, gender and with varying clinical status regarding insulin resistance. A clinical interview was conducted using questionnaires and basic laboratory parameters such as glucose and insulin concentration were measured with biochemical tests. The characteristics of these parameters are summarized in Table 4.

To select patients, the calculated homeostatic model assessment (HOMA-IR) was used, considering values of 2.5 and above as abnormal. The model used for calculations is presented below. On this basis, the group was divided into 2 groups: patients with insulin resistance (IR) consisting of 88 people and a control group of healthy patients (CTR) consisting of 112 people.

HOMA-IR = (Glucose [mmol/L] × Insulin [mU/mL])/22.5

### 4.2. Determination of Genotypes

DNA was isolated from blood collected from patients using a column-based extraction method. Each sample was analyzed for concentration and purity using a nanodrop. The following values were adopted as criteria for purity of the genetic material: for A260/A280 in the range of 1.8–2.0 and for A260/A230 in the range of 1.8–2.2. The extracted DNA was stored at −20 °C until further procedures.

Polymerase chain reaction (PCR) was performed in two separate batches for each of the single-nucleotide polymorphisms (SNPs). The commercially available reagent kit (Syngen Biotech, Wroclaw, Poland) was used for the reaction, containing, apart from polymerases, the final concentrations: MgCl_2_ 2.5 mM, dNTP mixture 200 μM each, BSA, genetic load and two dyes migrating in the gel—blue at approx. 4 kb and yellow at approx. 40 bp. The final volume of the reaction mixtures was 20 µL, the mastermix included the following components: 1 U of polymerase, 10 µM of each primer and 200 ng of genomic DNA.

#### 4.2.1. MMP-2 −1306 C/T (rs243865)

The following primers were used for the amplification reaction: sense primer 5′-CTTCCTAGGCTGGTCCTTACTGA-3′, antisense primer 5′-CTGAGACCTGAAGAGCTAAAGAGCT-3′.

The following PCR conditions were applied: activation at 95 °C for 15 min, followed by 35 cycles of denaturation at 95 °C for 20 s, annealing at 58 °C for 45 s, elongation at 72 °C for 1 min. The last stage of final elongation was performed at 72 °C for 10 min. The reaction products were cooled to 4 °C and stored for subsequent steps.

For the restriction fragment length polymorphism (RFLP), 10 µL of PCR products were incubated with the restriction enzyme FspBI (Thermo Scientific, Waltham, MA, USA) at 37 °C for 16 h. The enzyme was then inactivated by incubating for 20 min at 65 °C.

#### 4.2.2. MMP-9 −1562 C/T (rs3918242)

The following primers were used for the amplification reaction: sense primer 5′-GCCTGGCACATAGTAGGCCC-3′, antisense primer 5′-CTTCCTAGCCAGCCGGCATC-3′.

The following PCR conditions were applied: activation at 95 °C for 15 min, followed by 30 cycles of denaturation at 95 °C for 20 s, annealing at 58 °C for 1 min, elongation at 72 °C for 1 min. The last stage of final elongation was performed at 72 °C for 10 min. The reaction products were cooled to 4 °C and stored for subsequent steps.

For the RFLP step, 10 µL of PCR products were incubated with the restriction enzyme PaeI (Thermo Scientific) at 37 °C for 16 h. The enzyme was then inactivated by incubating for 20 min at 65 °C. The protocol conditions for genotyping patients using the PCR-RFLP method are presented in Table 5. The sequences of primers used in the method are summarized in Table 6.

Separation of the fragments was conducted by electrophoresis in a 3.5% agarose gel with the addition of nucleic acid gel stain at a voltage of 75 V for 60 min. As a control, PCR, digestion, and electrophoresis were performed for a blank sample in each batch. The following fragments were obtained for MMP-2 −1306 C/T (rs243865): 188 bp for the C allele and 162 bp + 26 bp for the T allele; for MMP-9 −1562 C/T (rs3918242): 435 bp for the C allele and 244 bp + 192 bp for the T alleles. The reading of individual fragments was performed in comparison with a DNA ladder template ranging from 700 bp to 25 bp (GeneRuler Low Range DNA Ladder, Thermo Scientific). Electrophoretic gel images after separation of MMP-2 and MMP-9 genes digestion products of some samples can be obtained in the Supplementary Materials.

### 4.3. Determination of MMP-2, MMP-9 and TIMP-1 Levels

MMP-2, MMP-9 and TIMP-1 concentration in the patients’ serum was determined using a commercially available quantitative enzyme-linked immunosorbent assay (#ab100606, #ab246539, #ab187394, Abcam, Waltham, MA, USA) according to the manufacturer’s instructions. Colorimetric reading was performed using a microplate reader.

### 4.4. Statistical Analysis

All statistical analyses were performed using JASP software (Version 0.95.0). To compare categorical variables of characteristics between the IR and CTR groups, the χ2 test was used, while the Kruskal–Wallis test was used for continuous variables within these groups. To precisely select the tests, the normality of data distribution was checked with the Shapiro–Wilk test, and the assessment of the equality of variances was checked with the Levene’s test. The relationship between genotypes and insulin resistance occurrence was evaluated using the adjusted odds ratios (OR) and corresponding 95% confidence intervals (95% CI). *p* < 0.05 was considered statistically significant. Furthermore, the Hardy–Weinberg equilibrium (HWE) was assessed by conducting a χ2 test comparing the observed genotype frequencies with the expected frequencies.

Descriptive statistics for MMP-2 and MMP-9 concentrations were performed separately for the IR and CTR groups. The associations between MMP-2 and MMP-9 levels and group assignment (IR vs. CTR) were assessed using the Mann–Whitney U test, preceded by the Shapiro–Wilk and Levene tests. The relationships between metalloproteinase concentrations and specific genotypes were evaluated using the Kruskal–Wallis test, whereas the analysis of metalloproteinase levels in relation to the presence of the T allele was conducted with the Mann–Whitney U test. Correlations between the concentrations of MMP-2 and MMP-9 and TIMP-1 levels were determined using Spearman’s rank correlation coefficient.

To evaluate the independent contribution of MMP-2 and MMP-9 polymorphisms to the risk of insulin resistance, a multivariate logistic regression analysis was performed using a Generalized Linear Model (GLZ). The model utilized a binomial distribution and a logit link function, with IR status as the dependent variable. The analysis was adjusted for potential clinical and metabolic confounders, including age, gender, BMI, triglycerides, and HDL. Qualitative predictors (genotypes and gender) were included using the following reference categories: CC genotype for MMP-2 and MMP-9, and male for gender. BMI and TG emerged as the only independent predictors of IR in the model. The model (Table 7) demonstrated that while BMI and triglycerides remained strong independent predictors of IR (*p* < 0.001), the associations with MMP-2 (rs243865) and MMP-9 (rs3918242) genotypes did not reach statistical significance after adjustment. These results suggest that the observed phenotypic differences in metabolic parameters exert a more dominant influence on IR status than the investigated genetic variants in this cohort.

Differences in TIMP-1 levels between healthy and insulin-resistant patient groups were divided into subgroups based on the set of alleles held, both for MMP-2 and MMP-9 SNPs. The Shapiro–Wilk test and Levene’s test were also performed for these data. The results from the studied patient groups were analyzed using Student’s *t* test for independent samples.

## 5. Conclusions

Within the limitations of our study cohort, no significant independent association was observed between the investigated MMP-2 and MMP-9 SNPs and the occurrence of insulin resistance. Importantly, these findings contribute to the ongoing discussion regarding the role of metalloproteinase-related genetic variability in metabolic disorders by providing additional population-specific evidence. Although previous studies have reported associations of these variants with type 2 diabetes and related metabolic complications, the present results suggest that their impact on insulin resistance may be indirect, context-dependent, or modulated by additional genetic and environmental factors.

Analyses of circulating metalloproteinase levels did not demonstrate a clear and consistent relationship with insulin resistance status; however, several observations merit further consideration. The tendency toward higher MMP-2 concentrations in the control group may point to a potential protective effect; nevertheless, this observation requires further investigation due to the limited number of comparable studies currently available. Likewise, the observed genotype-dependent variation in MMP-9 concentrations supports the known functional relevance of the rs3918242 polymorphism in regulating MMP-9 expression. At the same time, the lack of significant differences in overall serum MMP-9 levels between insulin-resistant and control groups suggests that mechanisms beyond total circulating concentrations may play a role in insulin resistance.

Taken together, the present findings highlight the complexity of metalloproteinase involvement in metabolic regulation. Given the multifactorial nature of insulin resistance, further studies conducted in larger and ethnically diverse populations are warranted to better elucidate the contribution of the analyzed polymorphisms and their interaction with other genetic and environmental determinants. In the present study, systemic TIMP-1 levels were not associated with IR status, regardless of the investigated genotypes. It is therefore important to replicate this study in larger, well-characterized cohorts to validate the observed trends and to clarify the potential clinical relevance of metalloproteinase-related pathways in insulin resistance.

## Figures and Tables

**Table 1:** Results of genotype and allele frequencies for individual groups.

SNP	IR	CTR	OR	*p*-Value
	*n* (%)	*n* (%)	(95% CI)	
MMP-2 −1306 C/T (rs243865)				
CC	54 (61.36)	64 (57.14)	Ref.	-
CT	31 (35.23)	43 (38.4)	1.1704 (0.6506–2.1047)	0.5993
TT	3 (3.41)	5 (4.46)	1.4063 (0.3212–6.1559) 0.7553 (0.1755–3.2504) *	0.6509 0.7063 *
CT + TT	34 (38.64)	48 (42.85)	1.1912 (0.6741–2.1050)	0.5470
Allele frequency				
C	139 (78.98)	171 (76.34)	Ref.	-
T	37 (21.02)	53 (23.66)	1.1644 (0.7235–1.8739)	0.5307
MMP-9 −1562 C/T (rs3918242)				
CC	61 (69.32)	84 (75.0)	Ref.	-
CT	24 (27.27)	23 (20.54)	0.6959 (0.3596–1.3467)	0.2818
TT	3 (3.41)	5 (4.46)	1.2103 (0.2786–5.2580) 0.7553 (0.1755–3.2504) *	0.7989 0.7063 *
CT + TT	27 (30.68)	28 (25.0)	0.7531 (0.4039–1.4042)	0.3724
Allele frequency				
C	146 (82.95)	191 (85.27)	Ref.	-
T	30 (17.05)	33 (14.73)	0.8408 (0.4903–1.4419)	0.5287

* values for CC + CT as ref. for TT genotype in calculation.

**Table 2:** MMP-2 concentrations in general groups and by MMP-2 −1306 C/T genotypes, and MMP-9 concentrations in general groups and by MMP-9 −1562C/T genotypes.

	IR	CTR	*p*-Value *
	[ng/mL]	[ng/mL]	
MMP-2			
General group	210.04 ± 41.46	225.85 ± 54.27	0.0227
MMP-2 −1306 C/T (rs243865) genotypes			
CC	209.24 ± 40.18	230.25 ± 58.59	0.0532
CT	198.31 ± 40.86	215.69 ± 49.89	0.2063
TT	245.71 ± 39.87	228.07 ± 61.64	0.3768
MMP-9			
General group	480.56 ± 191.99	469.59 ± 238.28	0.2411
MMP-9 −1562 C/T (rs3918242) genotypes			
CC	451.32 ± 171.54	453.18 ± 233.93	0.7349
CT	579.05 ± 228.73	482.32 ± 236.23	0.1552
TT	367.46 ± 49.64	556.80 ± 166.95	0.1116

* The *p*-value refers to the comparison of metalloproteinase concentrations between the groups of patients with insulin resistance (IR) and the control group (CTR) in the overall samples and by specific genotypes in the MMP-2 and MMP-9 polymorphisms.

**Table 3:** TIMP-1 concentration results for individual genotypes.

TIMP-1	IR	CTR	*p*-Value
	[ng/mL]	[ng/mL]	
MMP-2 −1306 C/T (rs243865)			
CC	271.52 ± 58.42	278.81 ± 64.09	0.6879
CT	297.07 ± 51.51	280.07 ± 44.77	0.5055
TT	283.62 ± 43.20	346.06 ± 119.97	0.5147
MMP-9 −1562 C/T (rs3918242)			
CC	290.22 ± 60.01	276.54 ± 57.84	0.7676
CT	255.43 ± 56.72	287.57 ± 70.54	0.3676
TT	252.57 ± 24.36	276.23 ± 55.30	0.3335

**Table 4:** Characteristics of parameters for individual study groups.

Characteristics	IR	CTR	*p*-Value
	*n* = 88	*n* = 112	
Age [years]	46.41 ± 16.92	43.91 ± 13.77	0.0289
Gender [female/male]	45/43	62/50	0.5525
Total cholesterol [mg/dL]	207.60 ± 38.95	206.80 ± 12.69	0.6702
HDL [mg/dL]	55.15 ± 15.83	60.60 ± 15.88	0.0185
LDL [mg/dL]	119.78 ± 40.92	122.08 ± 39.76	0.9764
Triglycerides [mg/dL]	154.35 ± 93.38	121.78 ± 71.91	0.0009
BMI [kg/m^2^]	29.38 ± 4.77	25.41 ± 3.99	<0.0001

**Table 5:** The protocol conditions for genotyping patients using the PCR-RFLP method for MMP-2 −1306 C/T (rs243865) and MMP-9 −1562 C/T (rs3918242) polymorphisms.

	MMP-2 −1306 C/T (rs243865)		MMP-9 −1562 C/T (rs3918242)	
1. Polymerase chain reaction (PCR)				
PCR Mix (per one reaction)		Gold Hot Start PCR MIX: 4 µL Forward primer: 0.6 µL Reverse primer: 0.6 µL PCR water: 12.8 µL DNA: 2 µL		Gold Hot Start PCR MIX: 4 µL Forward primer: 0.6 µL Reverse primer: 0.6 µL PCR water: 12.8 µL DNA: 2 µL
PCR conditions:		Activation: 15 min at 95 °C		Activation: 15 min at 95 °C
	35 cycles	Denaturation: 20 s at 95 °C	30 cycles	Denaturation: 20 s at 95 °C
		Annealing: 45 s at 58 °C		Annealing: 1 min at 58 °C
		Elongation: 1 min at 72 °C		Elongation: 1 min at 72 °C
		Final elongation: 10 min at 72 °C		Final elongation: 10 min at 72 °C
		Cooling: 4 °C		Cooling: 4 °C
2. DNA sequences restriction				
Reaction Mix		PCR product: 10 µL PCR water: 18 µL Tango Buffer: 2 µL FspBI Enzyme: 1 µL		PCR product: 10 µL PCR water: 18 µL Tango Buffer: 2 µL PaeI Enzyme: 1 µL
Restriction conditions		Incubation: 16 h at 37 °C		Incubation: 16 h at 37 °C
		Inactivation: 20 min at 65 °C		Inactivation: 20 min at 65 °C
3. Separation of the fragments by electrophoresis				
Agarose gel		Agarose: 3.5 g TBE Buffer 1X: 100 mL DNA Gel Stain: 5 µL		Agarose: 3.5 g TBE Buffer 1X: 100 mL DNA Gel Stain: 5 µL
Wells load		DNA: 10 µL + 2 µL Loading Buffer DNA Ladder: 6 µL		DNA: 10 µL + 2 µL Loading Buffer DNA Ladder: 6 µL
Electrophoresis conditions		50 V for 5 min 120 V for 120 min		50 V for 5 min 120 V for 120 min
Final products:		CC: 188 bp CT: 188 bp + 162 bp+ 26 bp TT: 162 bp+ 26 bp		CC: 435 bp CT: 435 bp + 244 bp + 192 bp TT: 244 bp + 192 bp

**Table 6:** Primers sequences for MMP-2 and MMP-9 polymorphism detection.

Genotype	Primer	Sequence
MMP-2 −1306 C/T (rs243865)	F primer	5′-CTTCCTAGGCTGGTCCTTACTGA-3′
	R primer	5′-CTGAGACCTGAAGAGCTAAAGAGCT-3′
MMP-9 −1562 C/T (rs3918242)	F primer	5′-GCCTGGCACATAGTAGGCCC-3′
	R primer	5′-CTTCCTAGCCAGCCGGCATC-3′

**Table 7:** Multivariate logistic regression analysis of factors associated with the risk of insulin resistance (IR).

	Odds Ratio	95% CI	*p*-Value
Age	0.98	0.96–1.00	0.1281
Gender			
male	Ref.		
female	1.24	0.63–2.44	0.5243
BMI	1.15	1.06–1.24	0.0003
Triglycerides	1.01	1.00–1.01	0.0022
HDL	1.01	0.99–1.04	0.3199
MMP-2 −1306 C/T (rs243865)			
CC	Ref.		
CT	0.99	0.53–1.84	0.5564
TT	1.61	0.41–6.38	0.4828
MMP-9 −1562 C/T (rs3918242)			
CC	Ref.		
CT	1.48	0.74–2.97	0.5369
TT	1.10	0.18–6.96	0.9169

## Data Availability

The data presented in this study are available on request from the corresponding author. The data are not publicly available due to privacy and ethical restriction.

## Supplementary Materials

The following supporting information can be downloaded at https://www.mdpi.com/article/10.3390/ijms27052158/s1.
