# Supplementary material for: MMP-2 and MMP-9 Gene Polymorphisms and Serum Levels in Relation to Insulin Resistance in a Polish Cohort
Source: Int J Mol Sci. 2026 Feb 25;27(5):2158. doi: 10.3390/ijms27052158 (PMC12984337; doi:10.3390/ijms27052158)
Supplement: Supplementary file 1 [file ijms-27-02158-s001.zip › ijms-4125618-supplementary.pdf]

Electrophoretic gel after separation of MMP-2 gene digestion products

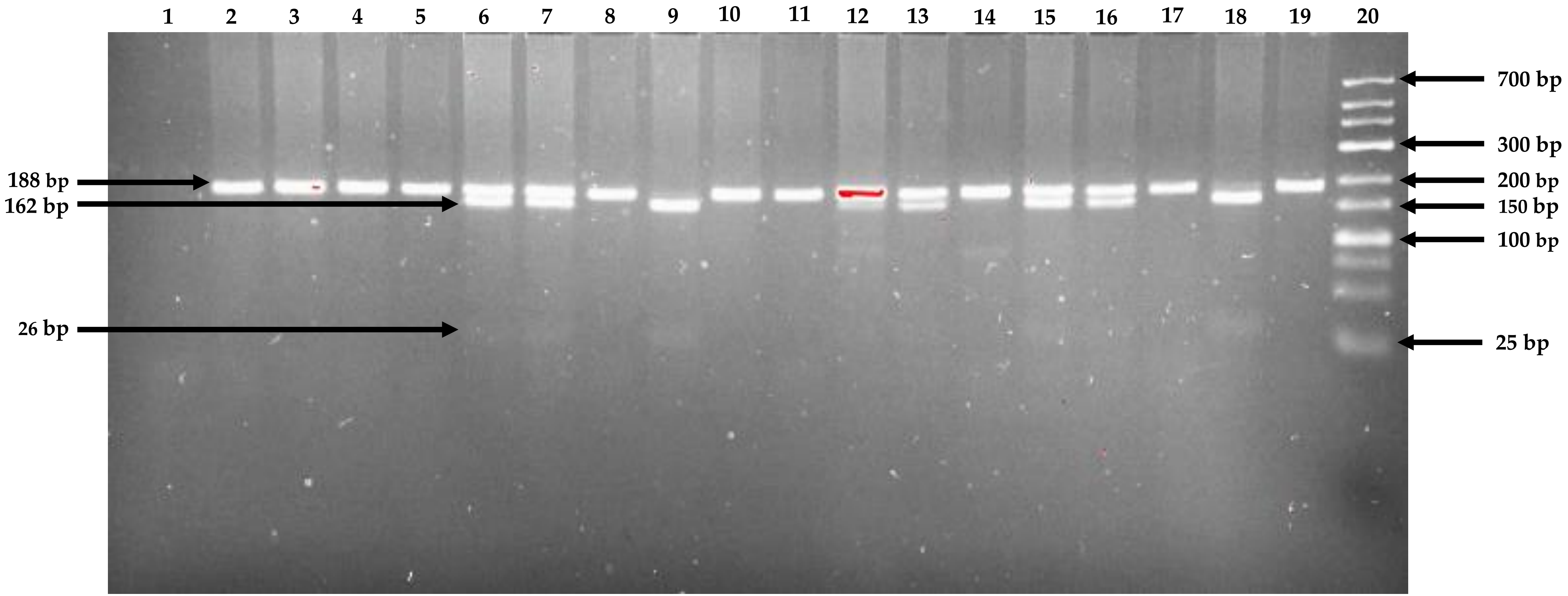

Lane 1: blank sample
Lane 2, 3, 4, 5, 8, 10, 11, 14, 17, 19: homozygous (CC) – 188bp
Lane 6, 7, 12, 13, 15, 16, 18: heterozygous (CT) – 188bp + 162bp + 26bp
Lane 9: homozygous (TT) – 162 bp + 26bp
Lane 20: DNA ladder

Electrophoretic gel after separation of MMP-9 gene digestion products

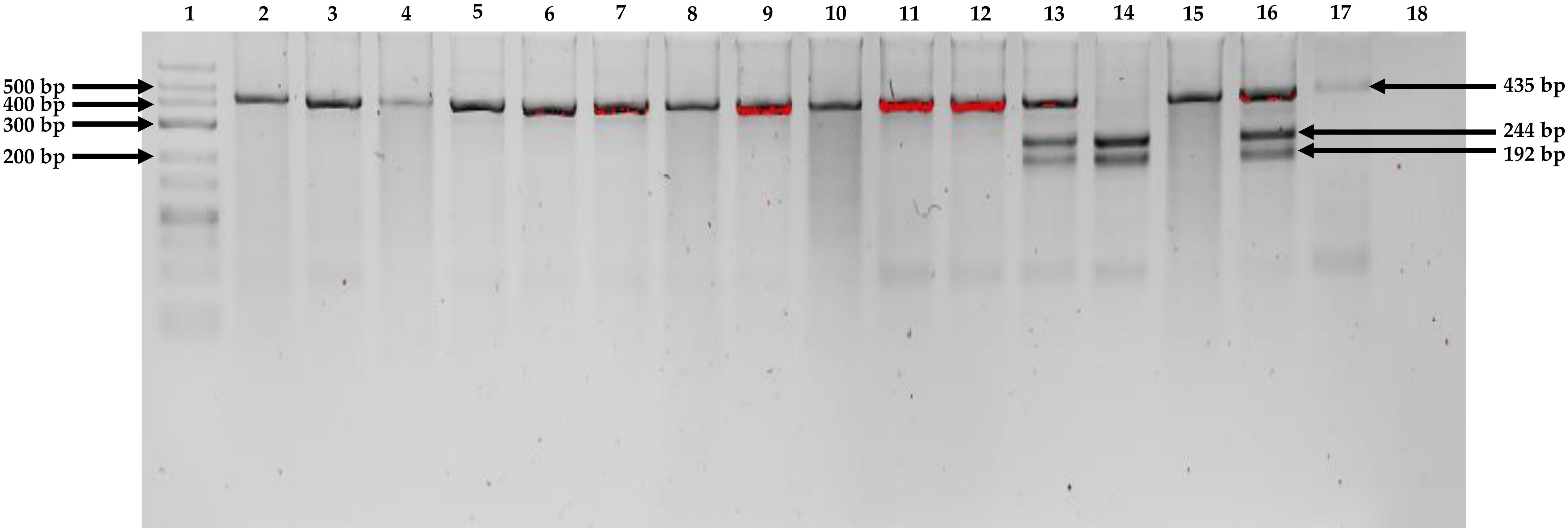

Lane 1: DNA ladder
Lane 2, 3, 4, 5, 6, 7, 8, 9, 10, 11, 12, 15, 17: homozygous (CC) – 435bp
Lane 13, 16: heterozygous (CT) – 435bp + 244bp + 192bp
Lane 14: homozygous (TT) – 244bp + 192bp
Lane 18: blank sample
